# Epithelial and Topographic Corneal Remodeling After Ptosis and Ectropion Surgery: An MS-39-Based Study

**DOI:** 10.3390/diagnostics16172758

**Published:** 2026-08-28

**Authors:** Maria Angela Romeo, Vanessa Ferraro, Alessandro Gaeta, Novella Montericcio, Giovanni Ottonelli, Silvia Ferraro, Giuseppe Giannaccare, Alessandra Di Maria

**Affiliations:** 1 Department of Ophthalmology, University Magna Graecia of Catanzaro, 88100 Catanzaro, Italy; 2 Ophthalmology Unit, AUSL-IRCCS di Reggio Emilia, Viale Risorgimento 80, 42123 Reggio Emilia, Italy; 3 Department of Internal Medicine and Medical Specialties (DIMI), Università Di Genova, 16132 Genova, Italy; 4 Biomedicine, Neuroscience and Advance Diagnostic (BIND) Department, University of Palermo, 90123 Palermo, Italy; 5 Department of Biomedical Sciences, Humanitas University, 20072 Milan, Italy; 6 Department of Ophthalmology, University of Catania, Via S. Sofia 78, 95123 Catania, Italy; 7 Eye Clinic, Department of Surgical Sciences, University of Cagliari, 09124 Cagliari, Italy; 8 Department of Ophthalmology, IRCCS Humanitas Research Hospital, 20089 Milan, Italy

**Keywords:** corneal epithelial thickness, eyelid malposition, ptosis, ectropion, anterior segment tomography, epithelial remodeling, corneal topography, refractive surgery screening, I-S asymmetry, SD-OCT

## Abstract

**Background/Objectives**: Eyelid malpositions can alter corneal shape through chronic mechanical forces, yet conventional topographic devices do not distinguish epithelial from stromal contributions to these changes. This study aimed to characterize layer-specific corneal remodeling after surgical correction of ptosis and ectropion using hybrid spectral-domain OCT/Placido tomography and to assess whether epithelial thickness mapping provides clinically relevant information beyond standard corneal topography. **Methods**: This study is a retrospective single-center study evaluating 33 eyes of 20 patients: 16 with ptosis and 17 with ectropion. MS-39 imaging was collected preoperatively, at 14 days, and at 6 months. Primary outcomes included sectoral epithelial thickness and inferior–superior (I-S) epithelial asymmetry. Secondary outcomes included central corneal and estimated minimum stromal thickness; keratometric values were analyzed as tertiary outcomes. **Results**: In the ptosis group, superior epithelial thickness increased from 40.7 to 44.3 µm at 6 months (*p* = 0.001), while I-S asymmetry decreased from 10.1 to 4.5 µm (*p* = 0.001). In the ectropion group, inferior epithelial thickness decreased from 54.8 to 51.0 µm, with changes detectable at 14 days, and I-S asymmetry decreased from 8.1 to 3.8 µm. Central corneal and estimated minimum stromal thickness remained stable in both groups. K2 and anterior corneal astigmatism decreased significantly by 6 months in both groups. **Conclusions**: Surgical correction of eyelid malpositions was associated with predominantly epithelial remodeling, while stromal thickness remained stable. These epithelial changes may modify keratometric and astigmatic measurements, supporting reassessment after corneal stabilization, the timing of which requires further characterization, in patients considered for refractive or premium cataract surgery.

## 1. Introduction

The corneal surface is continuously shaped by eyelid-generated mechanical forces. Approximately 10,000 blinks per day create repetitive shear stress and pressure on the corneal epithelium, mediated by the tear film, a non-Newtonian lubricant, and boundary lubricants such as lubricin [1,2]. The epithelium responds to these stimuli through mechanotransduction pathways involving TGF-β signaling and cytoskeletal reorganization [3,4,5]. In normal eyes, eyelid morphology directly influences corneal curvature and astigmatism, with palpebral fissure dimensions correlating significantly with corneal shape [6,7]. Disruption of ocular surface homeostasis by eyelid pathology or periocular surgery can induce iatrogenic complications, including tear film instability, conjunctival chemosis, epithelial damage, and postoperative ocular surface inflammation [8,9,10].

Ptosis, ectropion, and entropion each disrupt eyelid–cornea biomechanics through distinct pathophysiological mechanisms, compressive force in ptosis, exposure in ectropion, and mechanical abrasion in entropion, inducing different topographic alterations that are analyzed separately throughout this study. Chronic superior eyelid pressure in ptosis is associated with superior corneal flattening and with-the-rule astigmatism; ectropion causes exposure-related inferior irregularities [11,12]. While eyelid surgery may transiently alter the ocular surface microenvironment and confound immediate postoperative measurements, multiple studies confirm that surgical correction reverses these biomechanical changes: ptosis surgery reduces astigmatism and superior steepening, while lateral tarsal strip for ectropion improves inferior symmetry [11,13,14,15].

The corneal epithelium remodels its thickness profile to compensate for stromal irregularities and maintain optical quality [16]. In keratoconus, this produces the characteristic “donut pattern”, epithelial thinning over the cone apex with surrounding thickening [17,18]. This compensation means that identical anterior curvature maps may conceal fundamentally different stromal profiles [16,19]. This phenomenon, termed epithelial masking, refers to the capacity of the epithelium to redistribute its thickness in a manner that partially compensates for underlying stromal irregularities, thereby obscuring true stromal geometry on anterior surface measurements [16,20,21]. Standard devices measure total thickness and curvature but cannot differentiate epithelial from stromal contributions [22]. Advances in corneal regenerative medicine, including cell-based therapies for epithelial restoration, further underscore the clinical relevance of accurate epithelial assessment [5].

Prior studies of corneal changes after eyelid surgery relied exclusively on devices providing composite measurements [4,9,11,15]. While documenting curvature and pachymetric changes, they could not determine whether these represented stromal remodeling or epithelial compensation, a distinction with two potential clinical implications. First, epithelial thickness patterns may serve as biomarkers of mechanical stress relief in oculoplastic surgery [23,24]. Second, unrecognized epithelial masking can lead to misclassification of ectasia risk in refractive surgery screening, where epithelial mapping alters candidacy decisions in 7–19% of patients [25,26,27].

Hybrid platforms combining spectral-domain OCT (SD-OCT) with Placido disc topography now enable simultaneous acquisition of epithelial maps, stromal maps, and anterior curvature. This study aimed to characterize epithelial and stromal changes following surgical correction of ptosis and ectropion, determine the timing of corneal stabilization, and evaluate whether epithelial mapping provides clinically meaningful information beyond standard topography. The hypothesis was that eyelid surgery is associated with primarily epithelial remodeling rather than stromal changes and that epithelial patterns reveal mechanical stress relief invisible to conventional imaging.

## 2. Materials and Methods

**Study Design and Ethical Approval:** This study is a retrospective, observational, single-center study conducted at the Department of Ophthalmology, IRCCS Humanitas Research Hospital (Rozzano, Milan, Italy). The protocol adhered to the Declaration of Helsinki and was approved by the Institutional Ethics Committee (protocol code 4967, approved 15 July 2025). As this was a retrospective analysis of clinical data collected during routine care, all surgical procedures and imaging examinations were performed as part of standard clinical practice.

Participants: Consecutive patients who underwent surgical correction of eyelid malposition (ptosis or ectropion) at IRCCS Humanitas Research Hospital (Rozzano, Milan, Italy) between November 2024 and May 2025 were retrospectively identified for inclusion. Medical records and imaging data were reviewed between July 2025 and November 2025.

Inclusion criteria: adults (≥18 years) with involutional ptosis or ectropion requiring surgical correction, adequate fixation for imaging, and willingness to complete follow-up. Exclusion criteria: prior intraocular, refractive, or eyelid surgery; corneal pathology (keratoconus, pterygium, dystrophies, active surface disease); contact lens wear within 4 weeks; connective tissue disorders; inability to obtain artifact-free baseline imaging.

Surgical Procedures: All surgeries were performed by a single experienced oculoplastic surgeon (A.D.M.) using standardized techniques: (i) Ptosis: levator aponeurosis advancement (aponeurotic ptosis, levator function, ≥10 mm) or frontalis suspension with silicone rod (levator function ≤ 5 mm); (ii) Ectropion: lateral tarsal strip (LTS) for involutional ectropion with horizontal lid laxity; (iii) Entropion: LTS for involutional entropion (exploratory cohort; insufficient sample for formal analysis).

Postoperative care: tobramycin/dexamethasone four times daily for 2 weeks, followed by preservative-free artificial tears as needed.

### Imaging Protocol

Acquisition: Anterior segment imaging was performed using a hybrid spectral-domain OCT/Placido disc topography system (MS-39, CSO, Florence, Italy; 22,000 A-scans/s, axial resolution 3.5 µm, 22-ring Placido disc), enabling simultaneous acquisition of epithelial maps, stromal maps, and anterior curvature data [28].

All examinations were performed between 09:00 and 12:00 to minimize diurnal thickness variation [29]. Patients were instructed to blink twice immediately before acquisition to ensure adequate tear film distribution. Eyelids were gently retracted using a wire lid speculum (adjustable screw-type) without applying pressure to the globe. This standardized approach was adopted because eyelid malpositions, and particularly postoperative edema and chemosis in the early follow-up period, frequently impair spontaneous lid opening and compromise image acquisition quality. The speculum ensured consistent, artifact-free exposure of the corneal surface across all timepoints, eliminating both the variable mechanical effect of the pathological eyelid position and the operator-dependent variability of manual lid retraction. The same speculum type and retraction technique were used at every visit (baseline, 14 days, and 6 months) to maintain measurement consistency [30,31]. At least two consecutive scans were obtained at each visit; the highest-quality scan was selected.

Quality control: Placido ring continuity and centration verified; tear film artifacts identified by irregular ring patterns (examination repeated after preservative-free tears if present); epithelial segmentation quality confirmed in the central 6 mm zone; scans with persistent artifacts excluded. All assessments performed by a single trained examiner.

Timepoints: baseline (within 7 days preoperatively), 14 days (±3), and 6 months (±14) postoperatively. Intermediate timepoints (e.g., 1 and 3 months) were not systematically collected due to logistical constraints related to patient travel distance; however, when available, 3-month data were recorded and are reported as supplementary observations.

Outcome Parameters: The primary endpoint of the study was the change in inferior–superior epithelial asymmetry (I-S asymmetry), defined as inferior epithelial thickness minus superior epithelial thickness in the 3–6 mm annular zone, from baseline to 6 months after surgery. This parameter was selected a priori as the main outcome because it reflects the sectoral redistribution of epithelial thickness induced by eyelid-related mechanical forces and may provide a summary measure of corneal stress normalization after eyelid malposition correction.

Additional primary epithelial outcomes included central epithelial thickness, defined as average epithelial thickness within the central 3 mm zone, sectoral epithelial thickness in the 3–6 mm annulus superiorly, inferiorly, nasally, and temporally, and minimum and maximum epithelial thickness. Secondary outcomes included central corneal thickness, minimum total corneal thickness, and estimated minimum stromal thickness, calculated as minimum total corneal thickness minus minimum epithelial thickness. Because the two minima may not occur at the same corneal location, this parameter represents an approximation rather than a true point-by-point stromal measurement.

Tertiary outcomes included simulated keratometry at 3 mm, comprising K1 flat, K2 steep, and average keratometry, as well as anterior corneal astigmatism magnitude and axis.

All analyses were conducted separately for the ptosis and ectropion groups, reflecting their distinct pathophysiological mechanisms and the expectation of different remodeling patterns. Between-group comparisons were performed to characterize these differences quantitatively.

Statistical Analysis: Analyses used SPSS v29.0 (IBM Corp., Armonk, NY, USA). The primary analysis focused on the longitudinal change in I-S epithelial asymmetry from baseline to 6 months within each surgical group. Other epithelial, stromal, and topographic parameters were analyzed as secondary or exploratory outcomes. Normality was assessed using the Shapiro–Wilk test. Longitudinal changes (baseline, 14 days, 6 months) were evaluated with the Friedman test for non-parametric repeated measures; post hoc pairwise comparisons involved the Wilcoxon signed-rank test with Bonferroni correction (α = 0.017). Effect size: Cohen’s d. Between-group comparisons of change from baseline to 6 months (Δ6 m − BL) used the Mann–Whitney U test. Entropion (*n* = 4) was analyzed descriptively only. Bonferroni correction (α = 0.017) was applied only to post hoc within-group longitudinal comparisons. For all other analyses, including between-group comparisons, two-tailed *p* values < 0.05 were considered statistically significant.

Because both eyes were included when eligible, the primary adjusted inferential framework employed generalized estimating equations (GEEs) to account for inter-eye correlation. Non-parametric within-group tests (Friedman/Wilcoxon) are reported in parallel for transparency and comparability with the prior literature that used similar approaches. GEE models were fitted with patient as the clustering unit, an exchangeable working correlation structure, and Gaussian family distribution. Fixed effects included timepoint (baseline, 14 days, and 6 months), surgical group (ptosis vs. ectropion), and the timepoint-by-group interaction. As an additional robustness check, linear mixed-effects models with the same fixed effects structure and random intercepts for patient were also fitted. Entropion eyes were excluded from these adjusted models to preserve the primary ptosis-versus-ectropion comparison. As this was a retrospective study based on consecutive clinical cases rather than a prospectively planned enrollment, an a priori sample size calculation was not applicable to the study design; all eligible patients treated during the study period were included, and no comparable layer-specific epithelial variability data from hybrid SD-OCT/Placido platforms were available at the time to inform a target sample size in any case. The large effect sizes observed for the primary endpoint (Cohen’s d = 3.93 and 2.69 in the ptosis and ectropion groups, respectively) support the robustness of this specific finding, although the study remains underpowered for secondary and subgroup comparisons. Prospective studies should define sample size a priori based on a minimum clinically relevant effect size.

## 3. Results

Study Population: Of 52 screened eyes (44 patients), 37 eyes (22 patients) completed follow-up. The primary analysis included 33 eyes of 20 patients: 16 with ptosis (12 levator advancement, 4 frontalis suspension) and 17 with ectropion (LTS). Four entropion eyes (2 patients) were analyzed descriptively only and are reported separately. Mean age 70.2 ± 10.8 years; 54% female. Exclusions: incomplete follow-up (12) and imaging artifacts (3). No major complications were recorded; minor ecchymosis (6) and chemosis (4) resolved within 7 days. No patient required reoperation during the follow-up. Demographic and clinical characteristics are summarized in Table 1.

Within the ptosis group, frontalis suspension (*n* = 4) was reserved for eyes with poor levator function (≤5 mm), representing inherently more severe ptosis than those treated with levator advancement (*n* = 12, levator function ≥ 10 mm). Despite the difference in disease severity, both subgroups showed directionally consistent epithelial remodeling; however, given the small size of the frontalis suspension subgroup (*n* = 4) and the absence of formal statistical comparison, a technique-specific contribution to the magnitude of remodeling cannot be excluded, and the pooled ptosis results should be interpreted as reflecting the group as a whole rather than a single homogeneous surgical mechanism.

The primary analysis included the ptosis and ectropion groups (33 eyes, 20 patients). The entropion subgroup (4 eyes, 2 patients) was analyzed descriptively only.

Ptosis Group (*n* = 16): Epi S increased from 40.7 ± 0.8 to 44.3 ± 0.9 µm at 6 months (*p* = 0.001, d = 2.91); it was not significant at 14 days (*p* = 0.187). I-S asymmetry declined from 10.1 ± 1.0 to 4.5 ± 1.1 µm (*p* = 0.001, d = 3.93); it was significant at 14 days (*p* = 0.015, below corrected α = 0.017). Epi I decreased from 50.9 ± 0.5 to 48.9 ± 0.6 µm (*p* = 0.001, d = 2.41) and was already significant at 14 days (*p* = 0.011). Epi min increased from 38.8 ± 0.8 to 42.2 ± 1.1 µm (*p* = 0.001, d = 2.72). Epi avg 3 mm showed only a modest absolute increase (+0.3 µm). Epi N and Epi T remained unchanged (*p* > 0.05). CCT (542.4 ± 3.5 → 544.2 ± 2.8 µm; *p* = 0.455) and Est. Str-Thk min (500.6 ± 3.4 → 499.0 ± 3.2 µm; *p* = 0.442) were stable, confirming epithelial rather than stromal remodeling. K2 decreased from 45.0 ± 0.4 to 44.4 ± 0.4 D (*p* = 0.010, d = 0.93); CYL increased from −1.2 ± 0.2 to −0.8 ± 0.2 D (*p* = 0.002, d = 1.88); both were significant only at 6 months. K1 remained unchanged (*p* = 0.144). Relevant primary epithelial parameters are reported in Table 2.

Ectropion Group (*n* = 17): Epi I decreased from 54.8 ± 0.9 (higher than previously published normative values, ~51–52 µm; no concurrent control group) to 51.0 ± 0.5 µm (BL → 14 d *p* = 0.001; BL → 6 m *p* < 0.001, d = 4.01). I-S asymmetry declined from 8.1 ± 1.2 to 3.8 ± 0.8 µm (*p* < 0.001, d = 2.69). Changes were significant at 14 days. Epi S remained unchanged (*p* = 0.198). CCT (538.7 ± 4.1 → 540.8 ± 2.6 µm; *p* = 0.561) and Est. Str-Thk min (491.3 ± 3.9 → 493.2 ± 2.6 µm; *p* = 0.472) remained stable. K2 decreased from 45.3 ± 0.5 to 44.5 ± 0.4 D (*p* = 0.008, d = 1.04), detectable at 14 days. CYL increased from −1.2 ± 0.2 to −0.9 ± 0.2 D (*p* = 0.003, d = 1.21). K1 showed a significant Friedman trend (*p* = 0.002) but BL → 6 m *p* = 0.022 did not reach corrected α = 0.017. Table 3 summarizes longitudinal changes.

Entropion Subgroup (*n* = 4): Epi min increased from 41.2 ± 1.0 to 44.0 ± 0.0 µm (*p* = 0.022; SD = 0.0 reflects convergence of all four values in this small sample). I-S asymmetry decreased from 5.2 ± 1.3 to 2.3 ± 0.4 µm (*p* = 0.018; post hoc ns after correction). CCT and stroma remained stable. Given the insufficient sample size, these results are hypothesis-generating only and are not included in the primary analysis.

Between-Group Comparison: Between-group comparisons were restricted to the primary analysis cohort (ptosis vs. ectropion, 33 eyes). ΔEpi S: ptosis +3.45 (IQR 1.57) vs. ectropion +0.60 (0.90) µm (*p* < 0.001). ΔEpi I: −1.95 (1.15) vs. −4.20 (1.40) µm (*p* < 0.001). ΔI-S asymmetry was substantial in both groups (ptosis −5.70 [1.25]; ectropion −4.50 [2.20] µm; *p* = 0.018). ΔK2 (*p* = 0.460) and ΔCYL (*p* = 0.288) were comparable. Δ Est. Str-Thk min showed a borderline between-group difference (*p* = 0.034). Table 4 summarizes between-group comparisons.

**Figure 1:**
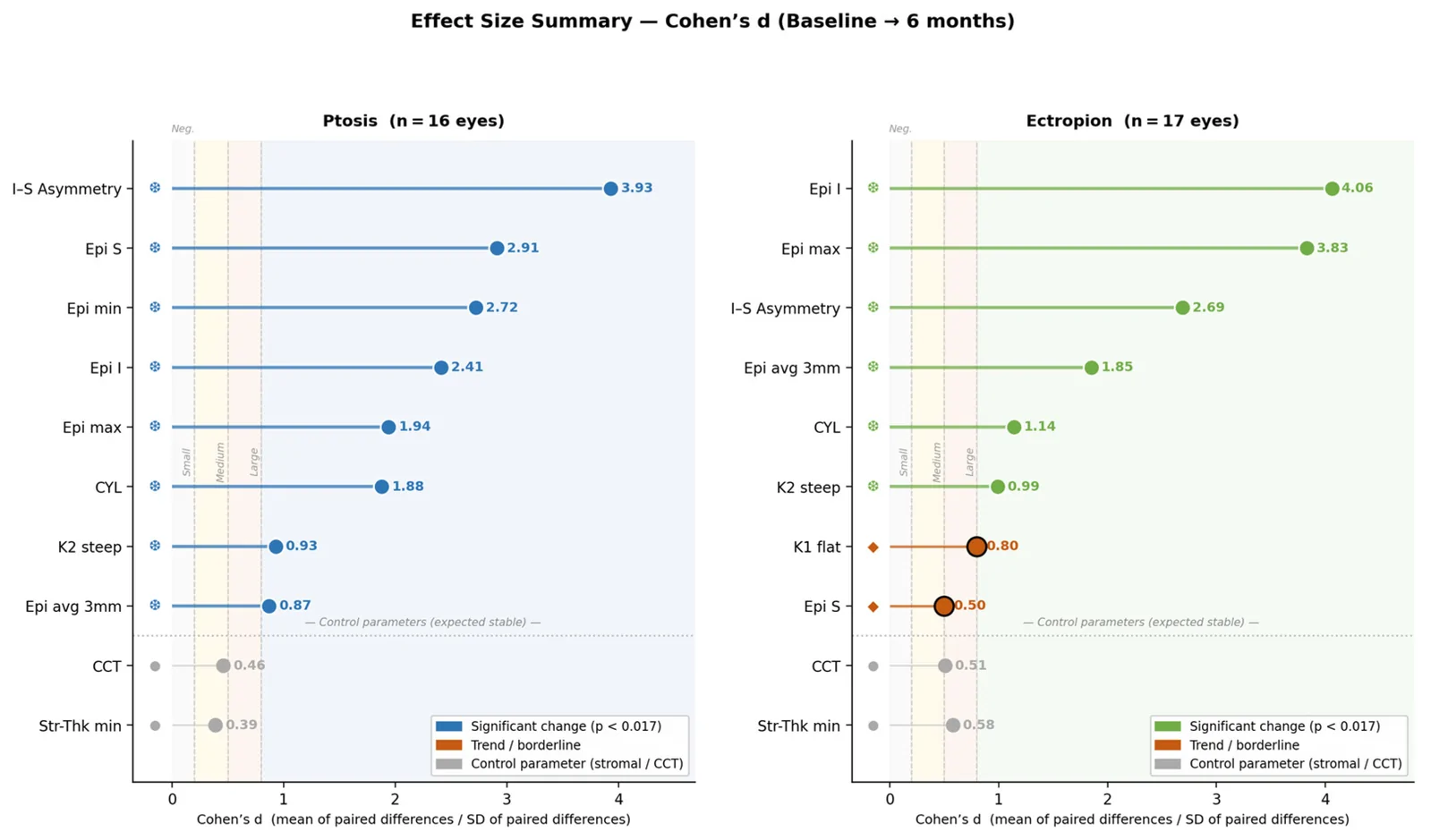
Cohen’s d effect sizes for all MS-39 parameters from baseline to 6 months postoperatively. Lollipop charts showing paired Cohen’s d for the ptosis group (**left**, n = 16 eyes) and ectropion group (**right**, n = 17 eyes). Effect sizes are color-coded by group (blue = ptosis; green = ectropion) for statistically significant changes (Bonferroni-corrected *p* < 0.017), orange for borderline or non-significant trends, and grey for control parameters expected to remain stable. Background bands indicate conventional thresholds: negligible (d < 0.2), small (0.2–0.5), medium (0.5–0.8), and large (>0.8); threshold boundaries are indicated by dashed vertical lines with zone labels. The dotted horizontal line separates epithelial outcome parameters (above) from stromal and total thickness control parameters (below). ✦ = statistically significant change; ◆ = borderline trend; ● = control parameter. d was calculated as the mean of paired differences divided by the standard deviation of paired differences. I–S = inferior minus superior epithelial thickness; Epi S/I = superior/inferior sectoral epithelial thickness (3–6 mm annulus); Epi min/max = minimum/maximum epithelial thickness; CCT = central corneal thickness; Str-Thk min = minimum stromal thickness (Thk min − Epi min); CYL = anterior corneal astigmatism magnitude; K2 = steep simulated keratometry.

Effect sizes for all MS-39 parameters are summarized in Figure 1.

Timing of Stabilization: Ptosis: Epi S not significant at 14 days; Epi I significant at 14 days (*p* = 0.011). Full significance at 6 months. Ectropion: epithelial and topographic changes significant at 14 days. Stroma stable at all timepoints in both groups.

Adjusted Analysis for Inter-Eye Correlation: GEE models, employed as the primary adjusted analysis to account for the non-independence of bilateral eyes, and confirmatory linear mixed-effects models accounting for inter-eye correlation and repeated measures yielded results fully consistent with the primary non-parametric analyses, confirming the robustness of the findings to intra-patient correlation. In the ptosis group, superior epithelial thickness increased significantly by 6 months, while I-S asymmetry decreased significantly, indicating progressive epithelial normalization. Inferior epithelial thickness also decreased. In the ectropion group, the time-by-group interaction revealed a distinct remodeling pattern, with greater inferior thinning and less superior thickening than in the ptosis group. Despite these anatomical differences, both groups showed marked reductions in I-S asymmetry. Stromal parameters showed no clinically meaningful changes. Central corneal thickness showed a minimal nominal change in the GEE models, but this finding was not consistently replicated in the linear mixed-effects analysis, supporting predominantly epithelial rather than stromal remodeling.

## 4. Discussion

Eyelid malpositions disrupt corneal biomechanics through chronic mechanical forces. Prior studies using Scheimpflug (Pentacam, OCULUS Optikgeräte GmbH, Wetzlar, Germany; or Sirius, CSO, Florence, Italy) and Placido (Keratograph, OCULUS Optikgeräte GmbH, Wetzlar, Germany) devices documented topographic changes, but the epithelial versus stromal contributions remained unknown. This study aimed to characterize layer-specific corneal remodeling after ptosis and ectropion surgery using hybridthe MS-39 (CSO, Florence, Italy), which combines SD-OCT with Placido topography.

### 4.1. Epithelial Remodeling Dominates over Stromal Changes

**Figure 2:**
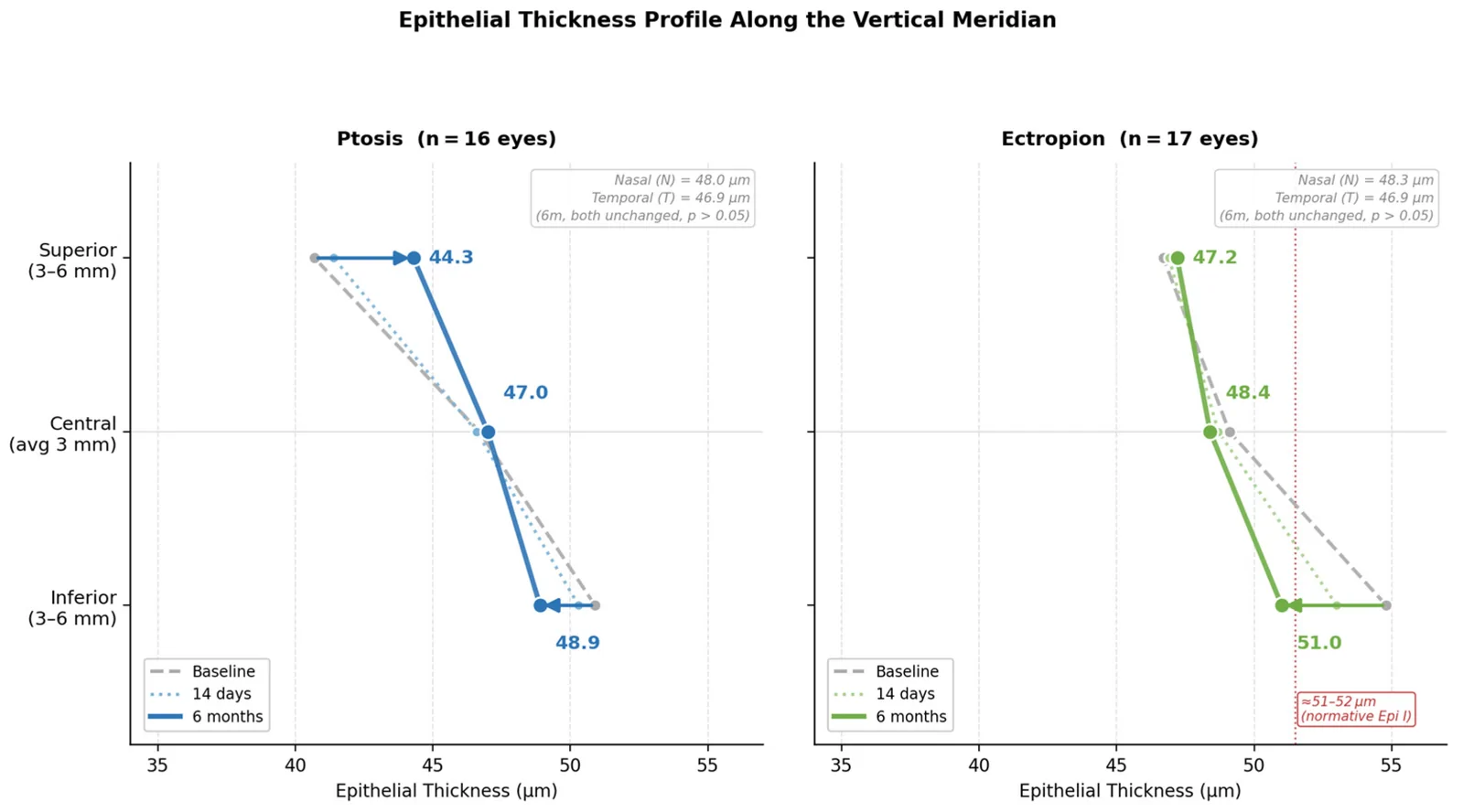
Epithelial thickness profile along the vertical meridian — baseline, 14 days, and 6 months. Line plots showing the epithelial thickness profile along the vertical axis for the ptosis group (**left**, n = 16 eyes) and ectropion group (**right**, n = 17 eyes). The y-axis represents anatomical position (inferior, central 3 mm average, superior); the x-axis represents epithelial thickness in micrometers. Each timepoint is represented by a distinct line style: dashed grey = baseline; dotted = 14 days; solid = 6 months (bold). Arrows on the superior and inferior segments indicate the direction of statistically significant change from baseline to 6 months. Nasal (N) and temporal (T) values at 6 months are reported in the inset box (upper right); these sectors did not change significantly in either group (*p* > 0.05). The dotted vertical reference line in the ectropion panel indicates the published normative range for inferior epithelial thickness (~51–52 µm). I–S = inferior minus superior epithelial thickness.

The principal finding is that surgical correction of eyelid malpositions was associated with predominantly epithelial remodeling while the stroma remained stable, a pattern observed consistently across both ptosis surgical techniques, although the pooled ptosis group combines two mechanistically distinct procedures and should not be interpreted as a single uniform intervention. In the ptosis group, superior epithelial thickening (+3.6 µm, d = 2.91) with concurrent I-S asymmetry reduction (10.1 → 4.5 µm, d = 3.93) occurred against a background of unchanged CCT and stromal thickness. This pattern is consistent with release of chronic mechanical compression from the drooping eyelid. The superior epithelium, which was initially thinner than previously published normative values obtained in independent cohorts (~51–52 µm; no concurrent control group was imaged in this study) (40.7 µm), thickened progressively after surgical decompression, while the inferior epithelium, which had compensatory thickening (50.9 µm), normalized downward (48.9 µm) [32]. After accounting for intra-patient correlation using GEE, the principal epithelial changes remained significant, whereas stromal thickness parameters showed no clinically meaningful postoperative variation. A small nominal CCT change observed in one GEE contrast was not consistently confirmed by mixed-effects sensitivity analysis and was below a magnitude likely to affect clinical interpretation. The progressive normalization of the vertical epithelial profile is illustrated in Figure 2.

Prior AS-OCT studies documented similar epithelial thickening after blepharoplasty for dermatochalasis and identified preoperative superior thinning in ptotic eyes [23,24]. The data extend these observations by demonstrating, using integrated layer-specific imaging for the first time in eyelid malposition surgery, that I-S asymmetry normalizes progressively over 6 months.

In the ectropion group, inferior epithelial normalization (54.8 → 51.0 µm, d = 4.01) is consistent with resolution of exposure-related compensatory thickening after restoration of lid–globe apposition. The baseline inferior epithelial thickness (54.8 µm) was higher than previously published normative values obtained in independent cohorts (~51–52 µm), and approached these external reference values by 6 months, although the absence of a concurrent control group precludes a direct statistical comparison [32]. Prior Orbscan studies documented postoperative steepening reduction and improved astigmatic regularity but could not identify the epithelial mechanism underlying these changes [11].

The divergent sectoral epithelial remodeling (superior thickening in ptosis, inferior thinning in ectropion) mirrors the opposite mechanical vectors of these conditions, confirming that they represent pathophysiologically distinct entities. Despite these different pathways, both groups achieved convergent normalization of I-S asymmetry toward physiological values (ptosis: 10.1 → 4.5 µm; ectropion: 8.1 → 3.8 µm), approaching, though not statistically compared against, the normative range of ~2–3 µm reported in independent healthy cohorts. This convergent normalization from divergent epithelial processes suggests that I-S asymmetry may serve as a candidate malposition-independent biomarker of corneal stress relief, analogous to its established role in ectasia screening [4,33].

### 4.2. Topographic Changes Reflect Epithelial Redistribution

**Figure 3:**
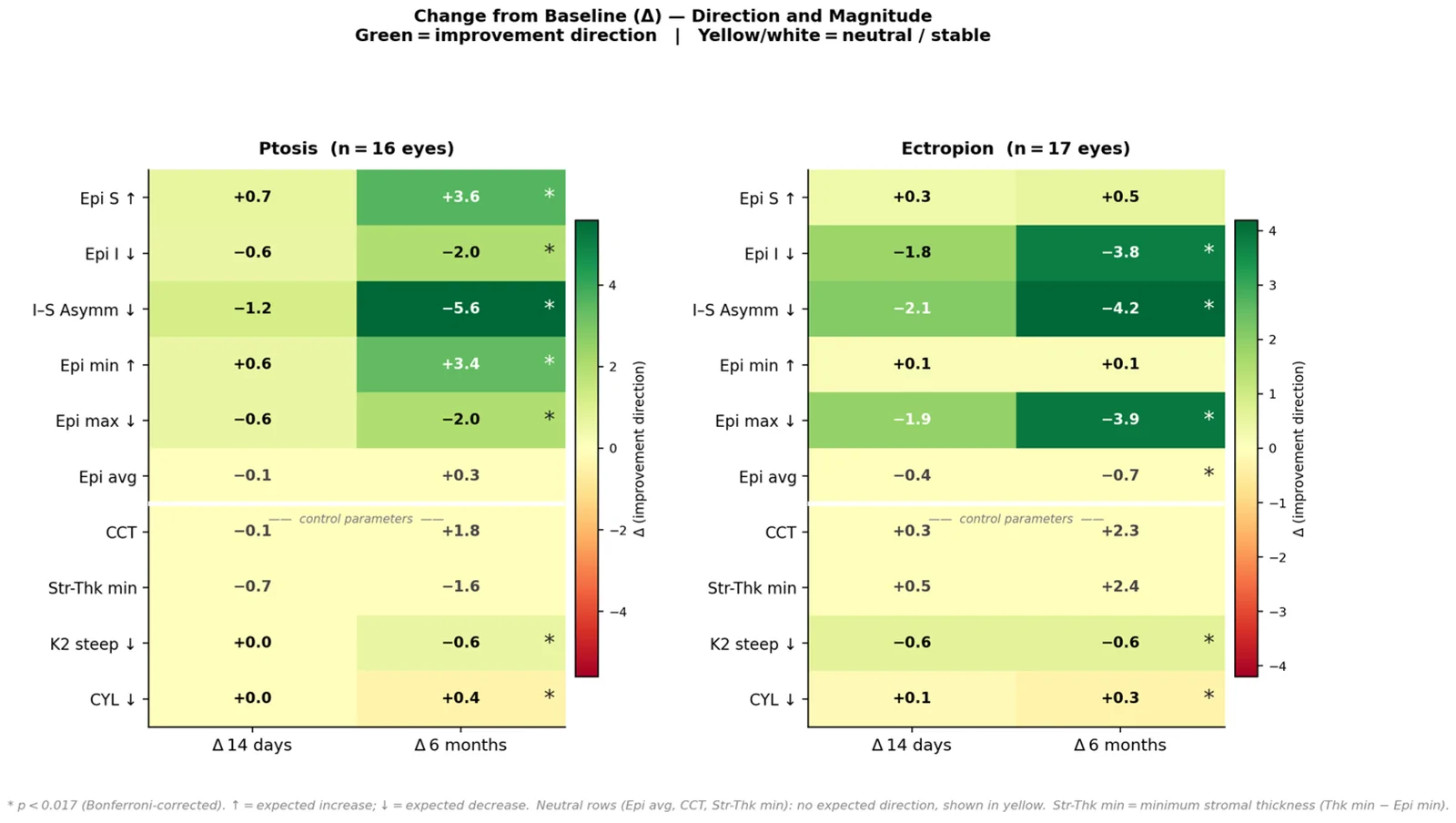
Change from baseline (Δ) in epithelial, stromal, and topographic parameters at 14 days and 6 months. Heatmaps showing the absolute change from baseline (Δ = postoperative value − baseline value) for all outcome parameters at 14 days and 6 months in the ptosis group (**left**, n = 16 eyes) and ectropion group (**right**, n = 17 eyes). Color encodes the direction and magnitude of change relative to the expected direction of clinical improvement: green = change in the expected direction of improvement; red = change opposite to the expected direction; yellow/white = negligible change or neutral parameter. Cell values represent absolute Δ in original units (µm or diopters); negative values are indicated by the minus sign (−, Unicode U+2212). Asterisks (*) indicate parameters reaching Bonferroni-corrected significance (*p* < 0.017) at 6 months. The white horizontal line labeled “control parameters” separates primary epithelial outcome parameters (above) from stromal and total thickness control parameters with no expected direction of change (below). Row labels indicate the expected direction of change after successful surgery (↑ increase; ↓ decrease). CCT = central corneal thickness; Str-Thk min = minimum stromal thickness (Thk min − Epi min); K2 = steep simulated keratometry; CYL = anterior corneal astigmatism magnitude; I–S = inferior minus superior epithelial thickness.

Concurrent K2 flattening (ptosis: −0.6 D; ectropion: −0.8 D) and CYL reduction (ptosis: +0.4 D; ectropion: +0.3 D) were observed in both groups, consistent with prior Pentacam and Sirius studies [14,15,28]. These findings suggest that topographic changes may be driven by epithelial redistribution rather than stromal remodeling. The stroma remained unchanged (ptosis: Est. Str-Thk min *p* = 0.442; ectropion: *p* = 0.472); the epithelium thickened or thinned to unmask the true stromal geometry previously obscured by compensatory redistribution. Standard topography alone would attribute these changes to stromal flattening, a different interpretation with different clinical implications [16,19]. The temporal pattern of epithelial versus stromal changes is visualized in Figure 3.

Prior imaging studies using Pentacam, Sirius, Orbscan, and Placido devices consistently reported curvature changes after eyelid surgery but could not differentiate epithelial from stromal contributions to postoperative curvature changes; hybrid SD-OCT/Placido platforms overcome this limitation through simultaneous layer-specific and topographic acquisition [23,24,34,35].

Not all prior studies have reported consistent topographic changes after eyelid surgery. Assadi et al. (2021) found no significant change in I-S asymmetry after congenital ptosis correction despite significant Steepest K reduction, and Kim et al. (2016) reported that 15–31% of eyes showed no measurable corneal curvature change after levator resection [35,36,37]. These discrepancies may reflect differences in ptosis etiology (congenital vs. involutional), surgical technique, imaging platform, or follow-up duration and underscore the need for standardized layer-specific imaging protocols.

### 4.3. The Masking Effect: Clinical Implications for Refractive Surgery and IOL Power Calculations

The epithelial compensation phenomenon appears to operate in eyelid-induced corneal changes, where identical anterior curvature maps may conceal different stromal profiles [16,17,18]. Eyelid surgery may therefore change corneal measurements through epithelial redistribution rather than stromal remodeling, meaning that postoperative changes in keratometry or astigmatism may reflect normalization of a previously compensated epithelial profile rather than true stromal shape changes.

This distinction may deserve consideration in patients being evaluated for procedures sensitive to keratometric accuracy. However, the present study did not include refractive, biometric, or toric IOL measurement data, and the magnitude of clinical impact, if any, of the observed epithelial changes on surgical planning remains to be quantified in dedicated studies. Patients with uncorrected eyelid malpositions may harbor unstable epithelial profiles that mask ectasia risk [25]. The AAO Corneal Ectasia Preferred Practice Pattern now recommends epithelial mapping for ectasia screening, and epithelial mapping alters refractive candidacy in 7–19% of cases [25,26,27,33]. The present data extend this principle to eyelid-induced mechanical corneal changes.

Regarding timing, epithelial changes in the ptosis group reached significance only at 6 months, while ectropion changes were detectable at 14 days. This difference likely reflects different mechanotransduction kinetics under compressive versus exposure stimuli. Prior studies have reported that ptosis surgery can alter keratometric values and IOL power by up to 0.55 D in severe cases and that blepharoplasty-induced astigmatic changes of 0.5–1.0 D can occur during the first 3 postoperative months [34,38,39]. Although the present study did not measure IOL power or refractive outcomes directly, these external data suggest that biometry performed during a period of active epithelial remodeling could plausibly yield keratometric values that do not reflect stabilized corneal geometry; whether this translates into a measurable effect on IOL selection in this population remains to be tested directly. The observation that epithelial remodeling in the ptosis group was still evolving between 14 days and 6 months raises the question of optimal timing for definitive corneal measurements after eyelid surgery. Prior recommendations suggest eyelid surgery should precede cataract surgery by at least 3 months, consistent with ASPS guidelines recommending 9–12 months for long-term outcome assessment; whether the epithelial remodeling documented here should influence this interval is an open question that requires studies incorporating serial biometric and refractive data [40]. Epithelial mapping may help identify ongoing remodeling and support postponing definitive biometry or refractive planning until corneal measurements have stabilized, with reassessment at approximately 6 months being reasonable in patients with preoperative eyelid malposition when keratometry, astigmatism, or epithelial maps are likely to influence surgical decision-making.

### 4.4. Mechanobiological Interpretation

The observed epithelial remodeling can be interpreted within a tribological and mechanotransduction framework [2]. Normal blinking maintains low-friction dynamics through boundary lubricants such as lubricin; eyelid malpositions disrupt this equilibrium through altered lid-wiper mechanics (ptosis) or exposure-related tear film instability (ectropion) [1]. Corneal epithelial cells respond to mechanical stimuli through TGF-β/SMAD2 signaling, cytoskeletal reorganization, and integrin-mediated mechanotransduction, pathways that likely mediate both the compensatory thickness redistribution observed preoperatively and the progressive normalization documented after surgical correction [3,4,41,42]. Environmental factors such as prolonged screen exposure may compound ocular surface stress [43]. The divergent temporal kinetics, rapid epithelial response in ectropion versus gradual normalization in ptosis, may reflect fundamentally different recovery processes: resolution of exposure-driven hyperproliferative signaling versus slow reversal of compression-induced epithelial remodeling. While this mechanistic framework is consistent with the observed data, direct molecular or cellular confirmation through in vivo confocal microscopy or tissue-level studies remains a priority for future investigation.

### 4.5. Limitations

This study has several limitations. Of the 52 eyes initially screened, 15 (29%) were excluded, primarily due to incomplete follow-up (*n* = 12) and imaging artifacts (*n* = 3). The sample size (33 eyes in the primary analysis, 37 including the exploratory entropion subgroup) is consistent with prior imaging studies in eyelid surgery and provided complete datasets at all three timepoints for every included eye but limits statistical power for subgroup analyses and correlations [23,24]. In addition, as this was a retrospective study, sample size was determined by clinical case availability during the study period rather than a pre-specified power calculation, a limitation inherent to the retrospective design, although the magnitude of the primary effect sizes mitigates concern about a false-positive result for the main endpoint. The estimated minimum stromal thickness was derived by subtracting minimum epithelial thickness from minimum total corneal thickness; however, these two minima may not correspond to the same corneal location. This parameter should therefore be interpreted as an approximation of stromal stability rather than a precise point-by-point measurement. While the consistency of this estimate across all timepoints and both groups supports the conclusion that stromal thickness did not change meaningfully, future studies should employ spatially co-registered stromal thickness maps to confirm this finding. Specifically, the ptosis group included two surgical techniques with potentially different biomechanical effects on the cornea. While descriptive data suggest consistent remodeling patterns across techniques, confirmation in larger technique-homogeneous cohorts is needed. The inclusion of both eyes from bilateral patients may have introduced intra-patient correlation, potentially affecting the assumption of independent observations in the primary non-parametric analyses. To assess the robustness of the findings, supplementary generalized estimating equation (GEE) models accounting for patient-level clustering and linear mixed-effects models were performed, and both yielded results consistent with the primary analyses. However, the relatively small number of patient clusters may have limited the precision of the GEE robust standard error estimates. The entropion subgroup (*n* = 4) was analyzed descriptively only and should be considered hypothesis-generating. No age-matched healthy control group was included. Epithelial thickness values were interpreted in the context of published normative data from MS-39 studies in healthy eyes (approximately 51–52 µm for the inferior sector), providing an external reference for the degree of postoperative normalization [32]. Nevertheless, the absence of a concurrent control group limits the ability to account for age-related, device-specific, or population-specific variability, and future studies should include an age-matched control cohort to strengthen these comparisons. Visual acuity, refractive data, and aberrometric profiles were not included in the present analysis. This was a deliberate methodological choice: the study was designed to isolate and characterize structural epithelial remodeling, a previously unmeasured phenomenon in eyelid surgery, without the confounding influence of functional outcome variability. The integration of structural and functional data, including best-corrected visual acuity and manifest refraction, represents a natural next step and a priority for future investigation aimed at establishing the clinical significance of the epithelial changes documented here. Epithelial parameters had stabilized by the 6-month assessment; however, the absence of systematic intermediate timepoints (months 1–3) means that the precise timing of stabilization cannot be determined and may have occurred earlier. When available, 3-month data were recorded and showed values intermediate between 14 days and 6 months in the ptosis group, but these observations were not systematically collected and should be interpreted with caution. Whether this epithelial stability persists beyond 6 months requires confirmation with extended observation (12–24 months). The routine use of a wire lid speculum during image acquisition, while ensuring standardized corneal exposure across visits, could theoretically influence tear film distribution or introduce subtle mechanical effects on the ocular surface. However, the Barraquer-type speculum was applied without globe pressure, and the same protocol was maintained at all timepoints, minimizing systematic bias. The hybrid SD-OCT/Placido platform used in this study is not universally available; however, the growing clinical adoption of layer-specific imaging and its recent inclusion in AAO ectasia screening recommendations suggest increasing accessibility in the near future [33]. Standardized ocular surface testing (tear film break-up time, Schirmer test, vital staining) was not performed in this protocol. The study was specifically designed to characterize layer-specific epithelial remodeling using hybrid tomography, and ocular surface evaluation, while clinically relevant, was beyond its intended scope and is being addressed in a dedicated parallel investigation. Nevertheless, subclinical ocular surface alterations could independently influence epithelial thickness measurements, and future studies integrating both structural and surface assessments would strengthen the interpretation of these findings. Systemic conditions, metabolic therapies, and chronic topical medications may influence ocular surface parameters and should be considered in future studies evaluating postoperative epithelial remodeling [44].

## 5. Conclusions

This retrospective single-center study suggests that surgical correction of eyelid malpositions is associated with predominantly epithelial remodeling, with superior epithelial thickening after ptosis correction and inferior epithelial normalization after ectropion repair, while stromal thickness parameters remained stable in this cohort. These findings support the potential value of hybrid SD-OCT/Placido tomography for distinguishing epithelial from stromal contributions to postoperative corneal changes, a distinction that conventional Scheimpflug, Orbscan, or Placido-only devices are not designed to provide [11,14,15,34].

The observed topographic changes may therefore be explained, at least in part, by epithelial redistribution rather than true stromal remodeling [16,19]. The reduction in I-S epithelial asymmetry through different anatomical patterns in ptosis and ectropion suggests that this parameter could represent a useful candidate marker of eyelid-related corneal stress relief, although this interpretation requires confirmation in larger and methodologically independent cohorts [33,45].

Epithelial changes after ectropion repair were detectable as early as 14 days, whereas in the ptosis group, they reached statistical significance by 6 months. The precise timing of stabilization could not be determined due to the absence of systematic monthly assessments and may have occurred earlier than 6 months. These data support a cautious approach when planning procedures that depend on stable corneal measurements, including refractive surgery and cataract biometry [34,38]. Pending further validation with refractive and biometric outcomes, a cautious approach, repeating corneal measurements after clinical stabilization rather than relying on a single preoperative assessment, may be reasonable in selected cases where postoperative epithelial remodeling could influence clinical decision-making.

The epithelial compensation observed in this cohort also suggests that uncorrected eyelid malpositions may contribute to variability in ectasia risk assessment and refractive screening. Rather than establishing a new standard of care, these findings extend the rationale for considering epithelial mapping in selected patients with eyelid-induced mechanical corneal changes, particularly when conventional topography and clinical findings are discordant. Hybrid platforms may provide additional information for both oculoplastic and refractive surgeons, but their routine perioperative use should be evaluated in future studies with larger samples, functional outcomes, and longer follow-up.

From a clinical standpoint, these findings suggest that eyelid malpositions should be considered a potential source of corneal measurement instability. In patients undergoing evaluation for refractive or cataract surgery, the potential influence of uncorrected eyelid malposition on corneal measurements warrants awareness, and repeat assessment after eyelid stabilization may be considered, although the clinical impact on surgical outcomes requires prospective validation. Hybrid epithelial mapping may help determine when the cornea has reached a stable postoperative state.

Overall, these results should be considered preliminary because of the limited sample size, single-center design, inclusion of both eyes in some patients, lack of an age-matched control group, and 6-month follow-up. Multicenter validation with larger cohorts, appropriate adjustment for inter-eye correlation, follow-up beyond 12 months, and correlation with ocular surface and visual quality measures will be necessary before epithelial mapping can be recommended as a standard perioperative tool in oculoplastic surgery.

## Figures and Tables

**Table 1:** **Demographics and Clinical Characteristics**. LF = levator function; LTS = lateral tarsal strip. Within the ptosis group, surgical technique was selected according to preoperative levator function, assessed as upper lid excursion from downgaze to upgaze with the frontalis muscle immobilized, in accordance with standard classification criteria. MRD1 was documented qualitatively in clinical records but was not measured with a standardized protocol suitable for quantitative analysis and is therefore not reported.

Characteristic	Ptosis (*n* = 16 Eyes)	Ectropion (*n* = 17 Eyes)	Entropion (*n* = 4 Eyes)
Patients, n	11	9	2
Age, years (mean ± SD)	68.2 ± 8.0	72.9 ± 5.6	72.5 ± 2.1
Female, n (%)	7 (64%)	4 (44%)	1 (50%)
Eyes (OD/OS)	11/5	9/8	2/2
Bilateral cases, n patients	5	8	2
Surgery type	Levator (LF ≥ 10 mm) 12, Frontalis (LF ≤ 5 mm) 4	LTS 17	LTS 4

**Table 2:** **Longitudinal changes in epithelial thickness, stromal thickness, and corneal curvature following ptosis surgery (*n* = 16 eyes).** Data are presented as mean ± SD at baseline and 6 months, with absolute change and *p*-values from Wilcoxon signed-rank post hoc tests (Bonferroni-corrected α = 0.017). Bold Δ values indicate statistically significant changes. The 14-day timepoint and non-significant parameters (Epi N, Epi T, Epi avg 3 mm, Epi max, K1) are omitted for clarity.

Parameter	Baseline (Mean ± SD)	6 Months (Mean ± SD)	Change (Δ)	*p*-Value
Epi S (µm)	40.7 ± 0.8	44.3 ± 0.9	**+3.6**	0.001
Epi I (µm)	50.9 ± 0.5	48.9 ± 0.6	**−2.0**	0.001
I-S Asymmetry (µm)	10.1 ± 1.0	4.5 ± 1.1	**−5.6**	0.001
Epi min (µm)	38.8 ± 0.8	42.2 ± 1.1	**+3.4**	0.001
CCT (µm)	542.4 ± 3.5	544.2 ± 2.8	+1.8	0.455
Est. Str-Thk min (µm)	500.6 ± 3.4	499.0 ± 3.2	−1.6	0.442
K2 steep (D)	45.0 ± 0.4	44.4 ± 0.4	**−0.6**	0.010
CYL (D)	−1.2 ± 0.2	−0.8 ± 0.2	**+0.4**	0.002

**Table 3:** **Longitudinal changes in epithelial thickness, stromal thickness, and corneal curvature following ectropion surgery (*n* = 17 eyes).** Data are presented as mean ± SD at baseline and 6 months, with absolute change and *p*-values from Wilcoxon signed-rank post hoc tests (Bonferroni-corrected α = 0.017). Bold values indicate statistically significant changes. The 14-day intermediate timepoint is omitted for clarity.

Parameter	Baseline (Mean ± SD)	6 Months (Mean ± SD)	Change (Δ)	*p*-Value
Epi I (µm)	54.8 ± 0.9	51.0 ± 0.5	**−3.8**	0.001
I-S Asymmetry (µm)	8.1 ± 1.2	3.8 ± 0.8	**−4.3**	0.001
Epi max (µm)	56.8 ± 1.0	52.9 ± 0.7	**−3.9**	0.001
CCT (µm)	538.7 ± 4.1	540.8 ± 2.6	+2.1	0.561
Est. Str-Thk min (µm)	491.3 ± 3.9	493.2 ± 2.6	+1.9	0.472
K2 steep (D)	45.3 ± 0.5	44.5 ± 0.4	**−0.8**	0.008
CYL (D)	−1.2 ± 0.2	−0.9 ± 0.2	**+0.3**	0.003

**Table 4:** **Comparison of 6-month changes (Δ) between ptosis (*n* = 16 eyes) and ectropion (*n* = 17 eyes) groups.** Data are presented as median (IQR) change from baseline to 6 months. Between-group comparisons were performed using the Mann–Whitney U test. Bold values indicate statistically significant differences (*p* < 0.05).

Parameter	Ptosis Δ	Ectropion Δ (Median, IQR)	*p*-Value
ΔEpi S (µm)	**+3.45 (1.57)**	+0.60 (0.90)	0.001
ΔEpi I (µm)	−1.95 (1.15)	**−4.20 (1.40)**	0.001
ΔI-S Asymmetry (µm)	**−5.70 (1.25)**	**−4.50 (2.20)**	0.018
ΔK2 steep (D)	−0.73 (0.76)	−0.85 (1.09)	0.460
ΔCYL (D)	+0.39 (0.29)	+0.35 (0.30)	0.288

## Data Availability

The data presented in this study are available upon request from the corresponding author due to ethical restrictions.

## Abbreviations

The following abbreviations are used in this manuscript:

AS-OCT	Anterior Segment Optical Coherence Tomography
BL	Baseline
CCT	Central Corneal Thickness
CYL	Cylinder (anterior corneal astigmatism magnitude)
Epi avg	Average Epithelial Thickness (central 3 mm zone)
Epi I	Inferior Epithelial Thickness (3–6 mm annulus)
Epi max	Maximum Epithelial Thickness
Epi min	Minimum Epithelial Thickness
Epi N	Nasal Epithelial Thickness (3–6 mm annulus)
Epi S	Superior Epithelial Thickness (3–6 mm annulus)
Epi T	Temporal Epithelial Thickness (3–6 mm annulus)
GEE	Generalized Estimating Equations
IOL	Intraocular Lens
IQR	Interquartile Range
I-S	Inferior minus Superior (epithelial asymmetry index)
K1	Flat Simulated Keratometry (3 mm zone)
K2	Steep Simulated Keratometry (3 mm zone)
LTS	Lateral Tarsal Strip
OD	Oculus Dexter (right eye)
OS	Oculus Sinister (left eye)
SD	Standard Deviation
SD-OCT	Spectral-Domain Optical Coherence Tomography
Est. Str-Thk min	Estimated Minimum Stromal Thickness (Thk min − Epi min)
Thk min	Minimum Total Corneal Thickness
TGF-β	Transforming Growth Factor Beta
AAO	American Academy of Ophthalmology
ASPS	American Society of Plastic Surgeons

## References

[1] Schmidt T.A., Sullivan D.A., Knop E., Richards S.M., Knop N., Liu S., Sahin A., Darabad R.R., Morrison S., Kam W.R. (2013). Transcription, Translation, and Function of Lubricin, a Boundary Lubricant, at the Ocular Surface. JAMA Ophthalmol..

[2] Lievens C.W., Rayborn E. (2022). Tribology and the Ocular Surface. Clin. Ophthalmol..

[3] Utsunomiya T., Ishibazawa A., Nagaoka T., Hanada K., Yokota H., Ishii N., Yoshida A. (2016). Transforming Growth Factor-β Signaling Cascade Induced by Mechanical Stimulation of Fluid Shear Stress in Cultured Corneal Epithelial Cells. Investig. Ophthalmol. Vis. Sci..

[4] Molladavoodi S., Robichaud M., Wulff D., Gorbet M. (2017). Corneal epithelial cells exposed to shear stress show altered cytoskeleton and migratory behaviour. PLoS ONE.

[5] Giannaccare G., Lixi F., Slidsborg C., Ozkan G., Gheorghe A.G., Arghirescu A.M., Namazbayeva A., Monfared M., Singh R.B., Jhanji V. (2026). Current Landscape and Future Prospects of Corneal Regenerative Medicine. Ophthalmol. Ther..

[6] Read S.A., Collins M.J., Carney L.G. (2007). The Influence of Eyelid Morphology on Normal Corneal Shape. Investig. Ophthalmol. Vis. Sci..

[7] Maseedupally V., Gifford P., Swarbrick H. (2015). Variation in Normal Corneal Shape and the Influence of Eyelid Morphometry. Optom. Vis. Sci..

[8] Di Maria A., Barone G., Gaeta A., Confalonieri F., Vinciguerra P., Vinci V., Klinger M., Ferraro V. (2024). Persistent Conjunctival Chemosis after Lower Lid Blepharoplasty: A Comparison of Different Surgical Techniques. J. Clin. Med..

[9] Romeo M.A., Taloni A., Borselli M., Di Maria A., Mancini A., Mollace V., Carnovale-Scalzo G., Scorcia V., Giannaccare G. (2025). Iatrogenic Ocular Surface Complications After Surgery for Ocular and Adnexal Tumors. Cancers.

[10] Foulsham W., Coco G., Amouzegar A., Chauhan S.K., Dana R. (2018). When Clarity Is Crucial: Regulating Ocular Surface Immunity. Trends Immunol..

[11] Detorakis E.T., Ioannakis K., Kozobolis V.P. (2005). Corneal Topography in Involutional Ectropion of the Lower Eyelid: Preoperative and Postoperative Evaluation. Cornea.

[12] Zhu T., Ye X., Xu P., Wang J., Zhang H., Ni H., Su Z., Ye J. (2017). Changes of corneal tomography in patients with congenital blepharoptosis. Sci. Rep..

[13] Ottonelli G., Celada Ballanti J., Gaeta A., Barone G., Montericcio N., Di Maria A. (2025). Upper Eyelid Static Surgical Approaches for the Treatment of Facial Palsy-Induced Lagophthalmos: A Systematic Review. J. Clin. Med..

[14] Savino G., Battendieri R., Riso M., Traina S., Poscia A., D’Amico G., Caporossi A. (2016). Corneal Topographic Changes After Eyelid Ptosis Surgery. Cornea.

[15] Acar Eser N., Serbest Ceylanoglu K., Sen E. (2023). Influence of Upper Eyelid Surgeries on Corneal Morphology Detected with Pentacam. Aesthetic Plast. Surg..

[16] Reinstein D.Z., Archer T.J., Vida R.S. (2022). Epithelial thickness mapping for corneal refractive surgery. Curr. Opin. Ophthalmol..

[17] Yang Y., Pavlatos E., Chamberlain W., Huang D., Li Y. (2021). Keratoconus detection using OCT corneal and epithelial thickness map parameters and patterns. J. Cataract. Refract. Surg..

[18] Yücekul B., Dick H.B., Taneri S. (2022). Systematic detection of keratoconus in OCT: Corneal and epithelial thickness maps. J. Cataract. Refract. Surg..

[19] Zhou W., Reinstein D.Z., Archer T.J., Nitter T., Feng Y., Mule G., Stojanovic A. (2022). The Impact of Epithelial Remodeling on Surgical Techniques Used in Topography-guided Surface Ablation in Irregular Corneas. J. Refract. Surg..

[20] Reinstein D.Z., Archer T.J., Gobbe M. (2013). Improved Effectiveness of Transepithelial PTK Versus Topography-Guided Ablation for Stromal Irregularities Masked by Epithelial Compensation. J. Refract. Surg..

[21] Hwang E.S., Schallhorn J.M., Randleman J.B. (2020). Utility of regional epithelial thickness measurements in corneal evaluations. Surv. Ophthalmol..

[22] Salomão M.Q., Hofling-Lima A.L., Lopes B.T., Canedo A.L.C., Dawson D.G., Carneiro-Freitas R., Ambrósio R. (2017). Role of the corneal epithelium measurements in keratorefractive surgery. Curr. Opin. Ophthalmol..

[23] Arslan N., Kocamış S.İ., Sabur H., Acar M. (2023). Evaluation of the Effect of Dermatochalasis and Upper Eyelid Blepharoplasty Surgery on Corneal Epithelial Thickness Alterations. Aesthetic Plast. Surg..

[24] Carreira P., Loureiro T., Carreira A.R., Gouveia-Moraes F., Cardoso A., Sampaio A., Campos P., Rodrigues-Barros S., Machado I., Campos N. (2023). The Effect of Upper Eyelid Blepharoplasty on Corneal Tomography and Epithelial Profile. Clin. Ophthalmol..

[25] Asroui L., Dupps W.J., Randleman J.B. (2022). Determining the Utility of Epithelial Thickness Mapping in Refractive Surgery Evaluations. Am. J. Ophthalmol..

[26] Mohr N., Alio Del Barrio J.L., Dirisamer M., Saad A., Kassumeh S., Mayer W.J., Priglinger S.G., Luft N. (2026). Inter-expert agreement on epithelial thickness mapping for refractive surgery screening: Variability highlights need for standardization. J. Cataract. Refract. Surg..

[27] Tañá-Rivero P., Orts-Vila P., Tañá-Sanz P., Ramos-Alzamora M., Montés-Micó R. (2024). Assessment of corneal epithelial thickness mapping by spectral-domain optical coherence tomography. Front. Med..

[28] Savini G., Schiano-Lomoriello D., Hoffer K.J. (2018). Repeatability of automatic measurements by a new anterior segment optical coherence tomographer combined with Placido topography and agreement with 2 Scheimpflug cameras. J. Cataract. Refract. Surg..

[29] Hashmani N., Hashmani S., Saad C.M. (2018). Wide Corneal Epithelial Mapping Using an Optical Coherence Tomography. Investig. Ophthalmol. Vis. Sci..

[30] Lieberman D.M., Grierson J.W. (2000). The Lids Influence on Corneal Shape. Cornea.

[31] Shaw A.J., Collins M.J., Davis B.A., Carney L.G. (2009). Eyelid Pressure: Inferences From Corneal Topographic Changes. Cornea.

[32] Kanellopoulos A.J., Asimellis G. (2013). In Vivo Three-Dimensional Corneal Epithelium Imaging in Normal Eyes by Anterior-Segment Optical Coherence Tomography: A Clinical Reference Study. Cornea.

[33] Jhanji V., Ahmad S., Amescua G., Cheung A.Y., Choi D.S., Lin A., Mian S.I., Rhee M.K., Viriya E.T., Mah F.S. (2024). Corneal Ectasia Preferred Practice Pattern^®^. Ophthalmology.

[34] Zinkernagel M.S. (2007). Effect of Upper Eyelid Surgery on Corneal Topography. Arch. Ophthalmol..

[35] Kim Y.K., In J.H., Jang S.Y. (2016). Changes in Corneal Curvature After Upper Eyelid Surgery Measured by Corneal Topography. J. Craniofacial Surg..

[36] Assadi F.A., Narayana S., Yadalla D., Rajagopalan J., Joy A. (2021). Effect of congenital ptosis correction on corneal topography- A prospective study. Indian J. Ophthalmol..

[37] Gandhi A., Mehta A., Naik M. (2020). Does Frontalis Sling Surgery for Congenital Ptosis Change the Corneal Topography and Refractive Characteristics Postoperatively?. Clin. Ophthalmol..

[38] Aydemir E., Aksoy Aydemir G. (2023). Ptosis effects on intraocular lens power calculation. J. Cataract. Refract. Surg..

[39] Brown M.S., Siegel I.M., Lisman R.D. (1999). Prospective Analysis of Changes in Corneal Topography After Upper Eyelid Surgery. Ophthalmic Plast. Reconstr. Surg..

[40] Kim K.K., Granick M.S., Baum G.A., Beninger F., Cahill K.V., Donnelly K.C., Kaidi A.A., Kang A.S., Loeding L., Loyo M. (2022). American Society of Plastic Surgeons Evidence-Based Clinical Practice Guideline: Eyelid Surgery for Upper Visual Field Improvement. Plast. Reconstr. Surg..

[41] Molladavoodi S., Kwon H.J., Medley J., Gorbet M. (2015). Human corneal epithelial cell response to substrate stiffness. Acta Biomater..

[42] Yang S., Zhang J., Tan Y., Wang Y. (2022). Unraveling the mechanobiology of cornea: From bench side to the clinic. Front. Bioeng. Biotechnol..

[43] Aragona P., Barabino S., Di Zazzo A., Giannaccare G., Villani E., Aiello F., Antoniazzi E., Bonini S., Cantera E., Carlini G. (2025). Dry Eye Disease: From Causes to Patient Care and Clinical Collaboration—A Narrative Review. Ophthalmol. Ther..

[44] Ferraro V., Gaeta A., Barone G., Confalonieri F., Tredici C., Vinciguerra P., Di Maria A. (2025). Proposing a New Classification for Managing Prostaglandin-Induced Enophthalmos in Glaucoma Patients. Clin. Ophthalmol..

[45] Sukhee N., Namba H., Ikeda M., Ichinohasama S., Kurihara T., Okamoto N., Yoshida J., Usui T. (2025). Corneal epithelium is altered in keratoconus and forme fruste keratoconus. Sci. Rep..

